# Supplementary material for: Reward System EEG–fMRI-Pattern Neurofeedback for Major Depressive Disorder with Anhedonia: A Multicenter Pilot Study
Source: Brain Sci. 2025 Apr 29;15(5):476. doi: 10.3390/brainsci15050476 (PMC12110136; doi:10.3390/brainsci15050476)
Supplement: Supplementary file 1 [file brainsci-15-00476-s001.zip › brainsci-3592915-supplementary.pdf]

Consensus on the Reporting and Experimental Design of clinical and cognitive-behavioural Neurofeedback studies (CRED-nf) best practices checklist 2020*

Appendix 1

Domain	Item #	Checklist item	Reported on page #
**Pre-experiment**			
	1a	Pre-register experimental protocol and planned analyses	N
	1b	Justify sample size	5
**Control groups**			
	2a	Employ control group(s) or control condition(s)	N
	2b	When leveraging experimental designs where a double-blind is possible, use a double-blind	N
	2c	Blind those who rate the outcomes, and when possible, the statisticians involved	N
	2d	Examine to what extent participants and experimenters remain blinded	N
	2e	In clinical efficacy studies, employ a standard-of-care intervention group as a benchmark for improvement	N
**Control measures**			
	3a	Collect data on psychosocial factors	8
	3b	Report whether participants were provided with a strategy	2
	3c	Report the strategies participants used	N
	3d	Report methods used for online-data processing and artifact correction	N
	3e	Report condition and group effects for artifacts	N
**Feedback specifications**			
	4a	Report how the online-feature extraction was defined	N
	4b	Report and justify the reinforcement schedule	3
	4c	Report the feedback modality and content	3
	4d	Collect and report all brain activity variable(s) and/or contrasts used for feedback, as displayed to experimental participants	N
	4e	Report the hardware and software used	2-3
**Outcome measures**			
Brain	5a	Report neurofeedback regulation success based on the feedback signal	N
	5b	Plot within-session and between-session regulation blocks of feedback variable(s), as well as pre-to-post resting baselines or contrasts	N
	5c	Statistically compare the experimental condition/group to the control condition(s)/group(s) (not only each group to baseline measures)	N
Behaviour	6a	Include measures of clinical or behavioural significance, defined a priori, and describe whether they were reached	8-14
	6b	Run correlational analyses between regulation success and behavioural outcomes	N
**Data storage**			
	7a	Upload all materials, analysis scripts, code, and raw data used for analyses, as well as final values, to an open access data repository, when feasible	N

*Darker shaded boxes represent *Essential* checklist items; lightly shaded boxes represent *Encouraged* checklist items. If a checklist item is "not addressed" in the manuscript, enter "N" in place of the page number. We recommend using this checklist in conjunction with the CRED-nf article, which explains the motivation behind this checklist and provides details regarding many of the checklist items.
